# Production of hollow-type spherical bacterial cellulose as a controlled release device by newly designed floating cultivation

**DOI:** 10.1016/j.heliyon.2018.e00873

**Published:** 2018-10-20

**Authors:** Toru Hoshi, Kazuyoshi Yamazaki, Yuki Sato, Takaya Shida, Takao Aoyagi

**Affiliations:** a Department of Materials and Applied Chemistry, College of Science and Technology, Nihon University, 1-8-14, Kanda-Surugadai, Chiyoda-ku, Tokyo 101-8308, Japan; b Graduate School of Science and Technology, Nihon University, 1-8-14, Kanda-Surugadai, Chiyoda-ku, Tokyo 101-8308, Japan

**Keywords:** Materials chemistry, Pharmaceutical chemistry, Natural product chemistry

## Abstract

We developed a novel cultivating system for hollow-type spherical bacterial cellulose (HSBC) gel production without any molds or template. It consisted of floating aqueous medium droplet containing *Gluconacetobacter xylinus* (*G. xylinus*) at the boundary of two non-mixed silicone oil layers. The fibrils of bacterial cellulose (BC) were produced at the interface of water and oil phases. Fibril layers effectively thickened layer-by-layer and eventually formed a shell structure. The size of the HSBC gel can be controlled by the volume of dropped cell suspension. For cell suspensions of 50 μL and 10 μL, HSBC gels of approximately 4.0 mm and 2.5 mm were obtained, respectively. The shell of the HSBC gel is the gelatinous membrane formed by well-organized fibril networks; they comprised type-I crystal structure of cellulose. Additionally, we studied release profile of the fluorescein isothiocyanate-dextran (FITC-Dex) and observed that it released rapidly from the HSBC gels compared to from the BC gels obtained by the static culture method. The release behavior from HSBC gel agreed satisfactorily with Higuchi model. Therefore, the shell of HSBC gel is surely a thin gelatinous membrane of BC, and would be useful as a drug release device.

## 1 Introduction

The most abundant renewable biopolymer is cellulose, a polysaccharide consisting of linear β-1,4-D-glucose units. Cellulose derivatives have many important applications in the fiber, paper, membrane, and polymer industries. Purified cellulose can be obtained from the isolation of plant cellulose or through the biosynthesis of different types of microorganisms, such as algae (*Vallonia* (Finkenstadt and Millane, 1998)), fungi (*Saprolegnia* (Frkaš, 1979)), and bacteria (*Acetobacter, Agrobacterium, Rhizobium*) (Matthysse et al., 1981; Napoli et al., 1975). Bacterial cellulose (BC) biosynthesized by *Acetobacter xylinum* was first discovered (Brown, 1886) and has been used in practical applications for several decades (Nishi et al., 1990; Okiyama et al., 1992; Gupta and Gupta, 2005; Watanabe et al., 1993). BC has good mechanical properties including tensile strength and modulus, high water-holding capacity, high porosity, high crystallinity, and good biocompatibility (Klemm et al., 2005). In addition to different microorganisms, pure BC is also produced by a cell-free system with improved structural, physico-mechanical, and thermal properties (Ullah et al., 2015, 2016).

To obtain BC, several culture methods can be applied using *Gluconacetobacter xylinus*; the most common method is static culture, which produces gelatinous cellulose mass at the interface between the air and the liquid culture media. In Indonesia and the Philippines, BC obtained by static culture is manufactured in large quantity as food called *Nata de coco*. However, its shape depends on that of the culture container.

Another method is agitated culture, using which, cellulose having a fibrous structure dispersed throughout the medium was synthesized (Chao et al., 2000; Watanabe et al., 1998). Under certain agitated culture conditions, *Acetobacter xylinum* strain NQ5 (ATCC 53582) produced isolated sphere-type cellulose (Czaja et al., 2004). Czaja et al. proposed that the cellulose ribbon was only produced at the surface of sphere-like celluloses, and the continuous shear force during agitation caused the cellulose ribbons to intertwine with each other to form the spherical structure.

As unique shape of the BC gel, tubular BC is reported by two different methods. In the first technique, a cylindrical glass matrix is immersed in a larger volume of the culture medium (Klemm et al., 2001, 2003). Tubular BC gel is produced at the air-culture medium interface that exists between the outer and inner matrices. In the static culture, a prolonged time of about 3–4 weeks is necessary for obtaining a thick BC gel of 20–30 mm (Yano et al., 2008); furthermore, the production of long tubular BC gel was difficult using this technique. The second technique uses polydimethylsiloxane (PDMS) as the mold of the BC gel (Bodin et al., 2007; Nimeskern et al., 2013). The PDMS, also called silicone, demonstrates a high oxygen penetration. Putra et al. (2008) reported a simple technique to biosynthesize tubular BC gel, and they could obtain tubular BC gel with desired length, inner diameter, and thickness. The tubular BC gel has excellent mechanical properties and its use as a vascular graft (Bäckdahl et al., 2011; Li et al., 2017) or soft tissue material (Lin and Dufresne, 2014) in medical and pharmaceutical applications was proposed.

Herein, we attempted to prepare the spherical BC gel by the formation of Water-in-Oil type droplets without any molds or template. The water phase contains *Gluconacetobacter xylinus* (*G. xylinus*) and culture medium, whereas, the oil phase can supply oxygen to support its culture. Preparation of spherical BC gels was predicted by culturing *G. xylinus* in medium droplets. Moreover, we hypothesized that it would be hollow-type spherical BC (as HSBC), that can have thin gelatinous membrane composed of cellulose networks, which would be produced at the interface of water and oil phases. Until now, to the best of our knowledge, there is no report about such HSBC gel and the culturing method. These HSBC gels are expected as a seamless capsule for drug delivery applications.

## 2 Experimental

### 2.1 Materials

Hestrine-Schramm's medium (HS medium) (Hestrin and Schramm, 1954) was used for incubation of the bacterial strain. It consisted of the mixture of 30 g D-glucose (Kanto Chemical Co. Inc.), 5.0 g mannitol (Kanto Chemical Co. Inc.), 5.0 g peptone (HIPOLYPEPTONE^TM^, Nihon Pharmaceutical Co. Ltd.), 5.0 g Bacto^TM^ yeast extract (BD Biosciences), and 1.0 g magnesium sulfate heptahydrate (MgSO_4_·7H_2_O; Kanto Chemical Co. Inc.) in 1000 mL MilliQ water. Fluorescein isothiocyanate-labeled dextran (FITC-Dex, 10,000 g/mol) was purchased from Merck Co. Silicone oils (KF-56 and KF-54) were obtained from Shin-Etsu Chemical Co., Ltd. Other reagents were purchased from Kanto Chemical Co. Inc., and used as-received.

### 2.2 Preparation of hollow-type spherical BC gels

**Fig. 1:**
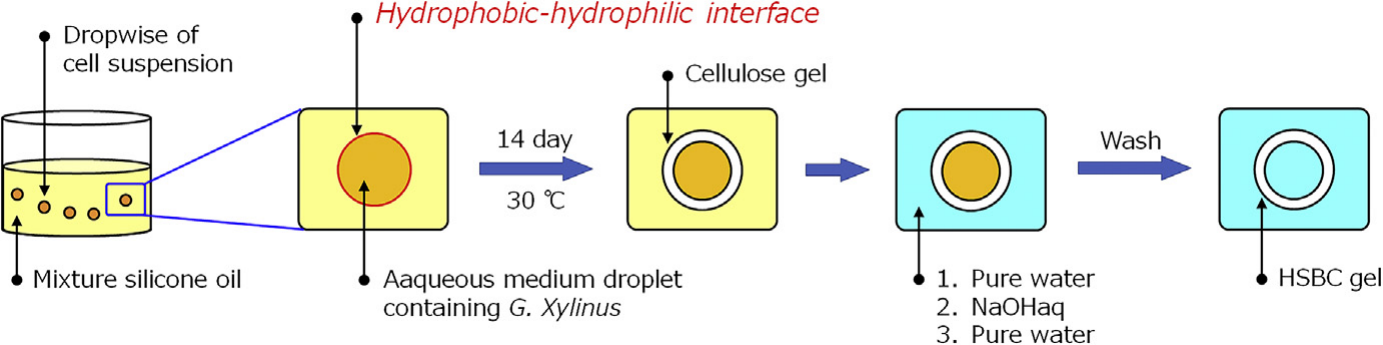
Schematic representation of production of the HSBC gel.

Fig. 1 shows the schematic representation of production of the HSBC gel. The HS medium was sterilized by autoclaving and *G. xylinus* (IFO13772) was cultured in the HS medium at 30 °C for 3 days. The cultured cell suspension was diluted with the same medium and dropped aseptically into 100 mL mixture silicone oils (Volume ratio; KF-56:KF-54 = 1:1) and incubated at 30 °C for 14 days by keeping the droplets floating.

The droplet size was controlled by the amount of drop (10–50 μL). The HSBC gel thus obtained was purified by soaking in a large quantity of distilled water for 1 day followed by washing in a 1% (w/v) aqueous solution of NaOH at room temperature for 1 day to remove the bacterial cell debris and alkali-soluble components. Then, it was washed several times with large quantity of distilled water and stored in distilled water at room temperature.

As a comparative sample, the conventional BC gel obtained by the static culture method. *G. xylinus* were grown under static conditions in a glass test tube (ID 14.5 mm, OD 16.5 mm, and height 165 mm, respectively) at 30 °C for 14 days. First, the solution became turbid, and a BC gel appeared on the air-culture medium interface. The gel thickness increased gradually, reaching 10–20 mm after 14 days. Purification of conventional BC gel was carried out by the same method as the HSBC gel.

### 2.3 Preparation of HSBC aerogel using supercritical CO_2_

After HSBC gel swelled in water, it was put in a large quantity of methanol and washed thoroughly and swelling solvent was changed from water to methanol completely. The gel was dried by supercritical CO_2_ (scCO_2_) technique without disintegrating its microstructure (Buchtová and Budtova, 2016). The drying was carried under conditions of 40 °C, 20 MPa, CO_2_ flow rate 2.0 mL/min, and 5 h. The drying apparatus consisted a CO_2_ delivery pump (SCF-Get, JASCO, Japan), 50 mL pressure vessel, a gas pressure regulator (SCF-Bpg, JASCO, Japan), and a constant temperature water bath (BK33, Yamato Scientific Co. Ltd., Japan).

### 2.4 Characterization of HSBC gels

The microstructure of HSBC aerogels was observed using a field-emission scanning electron microscope (FE-SEM: Hitachi High-Technologies Corporation S-4500) with an acceleration voltage of 10 kV. For the pretreatment to FE-SEM observation, deposition of Pt-Pd was performed by ion sputtering (Hitachi High-Technologies Corporation E-1010).

To analyze the composition and the crystal structure of the obtained HSBC aerogels, wide angle X-ray diffraction (WAXD) and attenuated total reflection Fourier transform infrared (ATR-FTIR) measurement were carried out. WAXD experiments were performed at 20 °C using an X-ray diffractometer (PANalytical X'Pert PRO MPD). The Cu-Kα radiation (wavelength, λ = 0.154 nm) was generated at 40 kV and 200 mA. The sample was scanned at a rate of 3°/min between 10° and 40° in transmittance mode. The ATR-FTIR spectra were measured using a FTIR spectrophotometer (Perkin Elmer Spectrum One) equipped with universal ATR sampling accessory. All measurements were carried out with a nominal spectral resolution of 1 cm^−1^ in transmittance mode and 24 scans.

### 2.5 Drug loading and release study

**Fig. 2:**
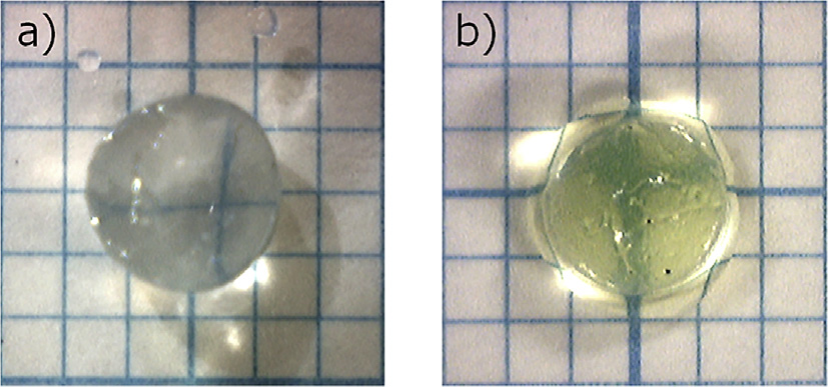
Photographs of a) HSBC gel and b) FITC-Dex loaded HSBC gel.

To evaluate the drug release behavior from the obtained HSBC gel, FITC-Dex (Mw = 10,000) was used as the model drug. The HSBC gel was immersed in a 1 mg/mL FITC-Dex aqueous solution and kept in a dark place at 4 °C for one day, and permeation of FITC-Dex into the HSBC gel was observed (Fig. 2). The release behavior of FITC-Dex from within the HSBC gel was evaluated by UV-Vis spectrometer (JASCO Corporation, V-530). The absorbance at the fixed wavelength of 490 nm was measured every 10 seconds at 25 °C continuously under stirring conditions at 500 rpm.

## 3 Results and discussion

### 3.1 Formation of cell suspension droplets

**Fig. 3:**
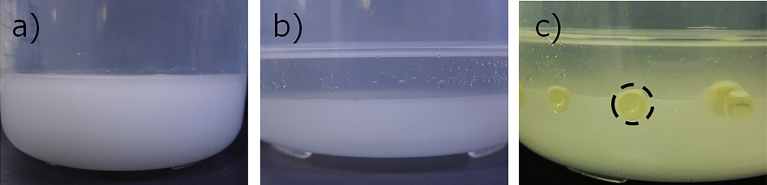
Photographs of a) mixed silicone oil immediately after mixing, b) phase-separated silicone oil, and c) formed cell suspension droplets.

To form the floating cell suspension in silicone oil phase, at first, the density of the cell suspension at 30 °C was measured to be 1.02 g/cm^3^ using the Baume hydrometer. Since silicone oil with the same density is not commercially available, we tried to adjust the density of silicone oil by two methods. The first method was to mix silicone oil and a miscible organic solvent having a density of >1 g/cm^3^ such as *o*-dichlorobenzene. In this method, however, although density control of the silicone oil was easy, the organic solvent inhibited the growth of *G. xylinus*, and hence, cellulose was not produced. The other was density adjustment by mixing silicone oils which have different specific gravities. Essentially, the two kinds of silicone oils, KF-56 (0.96 g/cm^3^) and KF-54 (1.07 g/cm^3^), are used in this method. Although these oils were immiscible and become cloudy when mixed (Fig. 3a), after they were left undisturbed, they separate into a transparent phase and a white turbid phase (Fig. 3b). When the cell suspension was added to the separated oil, spherical droplets were formed at the transparent-turbid phase interface (Fig. 3c). These droplets moved easily by slight shaking and fused into larger droplets by contact. To control HSBC gel size, it was necessary to suppress the contact between the droplets. Therefore, we focused on the kinematic viscosity of silicone oil. KF-56 with different kinematic viscosities are available. The same experiment was carried out using the KF-56 of 100, 350, and 500 cps; at a low kinematic viscosity of 100 cps, the phase separation speed was higher, and hence, contact between the droplets occurred frequently. On the contrary, at 350 and 500 cps, it was possible to slow down the phase separation and suspend the droplets at the phase interface for 14 days with a good stability.

### 3.2 Preparation of hollow-type spherical BC gels

**Fig. 4:**
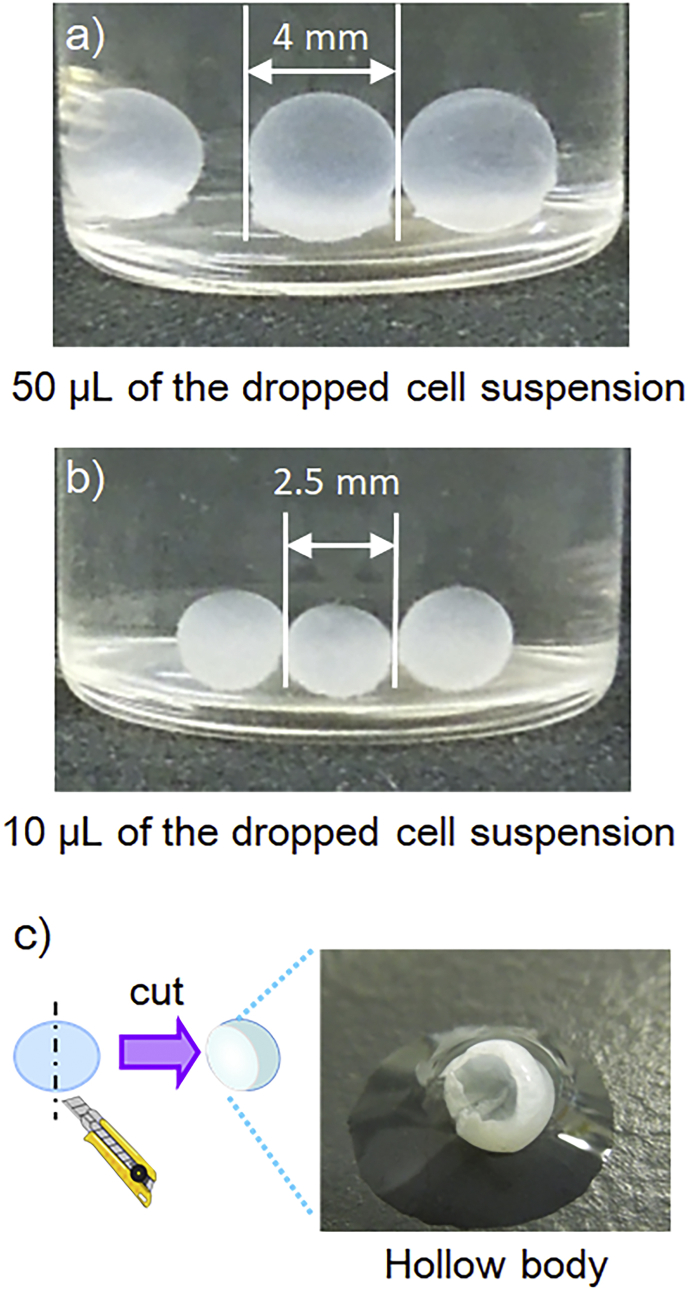
Photographs of hollow-type spherical BC gels.

Fig. 4 shows the result after 14-day culture by using a mixture of KF-56 (350 cps) and KF-54 oils. The size of the HSBC gel can be controlled by the volume of dropped cell suspension. For cell suspensions of 50 μL and 10 μL, HSBC gels of approximately 4.0 mm and 2.5 mm were obtained, respectively. In the static culture using test tube, the cellulose gel membrane produced at the air-medium interface sank gradually and cellulose was always produced at the air-medium interface. Hence the film thickness of the pellicle increased. When the cell suspension used in this study was static-cultured at the air-liquid interface for 14 days, the thickness of the pellicle was >1 cm. On the other hand, the thickness of the HSBC gel was extremely low (Fig. 4c), despite culturing for 14 days. This was due to the fact that the droplets were small, the amount of glucose was less, and hence, the amount of cellulose produced was eventually less. The direction of growth of the cellulose gel film was considered to be another factor. Unlike the static culture using test tube, in the suspended droplet culture, since cellulose was produced at the silicone oil-culture interface, the produced cellulose gel film grew towards the center of the droplet. The swelling cellulose gel film contracting towards the center, where the radius decreases, is unlikely. Therefore, it can be inferred that the thickness of the cellulose gel film would not increase. By extension of the culture duration, the cellulose fiber density, rather than the thickness of the gel film, is likely to increase.

### 3.3 Structural analysis of the HSBC gel after supercritical drying

**Fig. 5:**
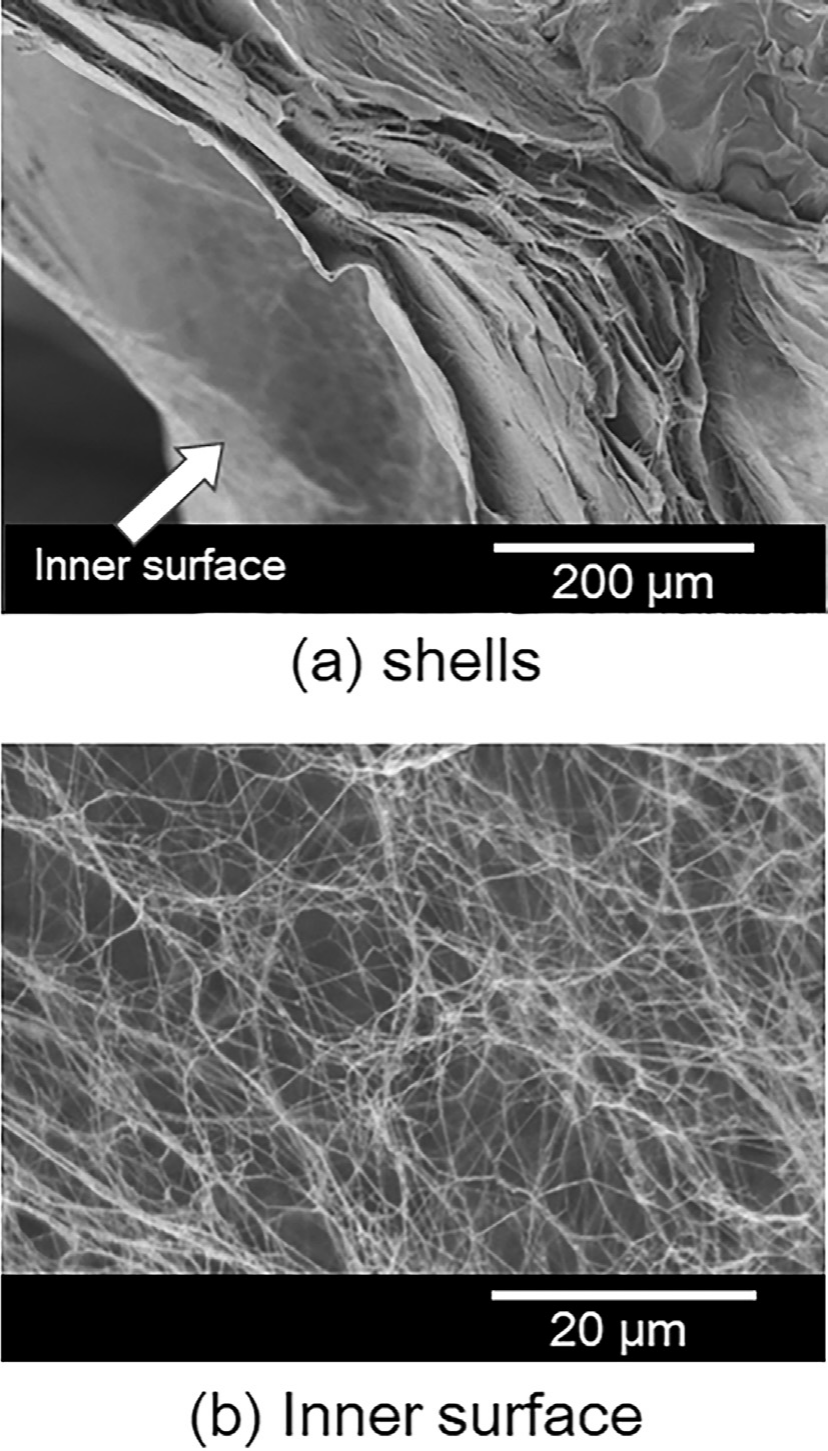
Scanning electron microscopy images of hollow-type spherical BC aerogel.

Fig. 5 shows the scanning electron microscopy (SEM) image of a BC gel aero-gelled by scCO_2_ drying. The cross-sectional structure of the HSBC gel film was found to be a multilayered structure (Fig. 5a) and the inner surface was found to be a network formed by cellulose microfibrils (Fig. 5b). Since *G. xylinus* produced cellulose microfibrils while undergoing repeated cell division, they form a fine in-plane microfibril network. In static culture, since this production activity took place on the surface of the medium (air-medium interface), microfibril networks were produced layer by layer, thus forming a laminated structure that increased in thickness (Staiger et al., 2007). The cellulose in the HSBC gel formed a gelatinous membrane composed of microfibril networks at the silicone oil-culture interface in the same way as cellulose production at the air-medium interface, thus forming a laminated gelatinous membrane structure.

**Fig. 6:**
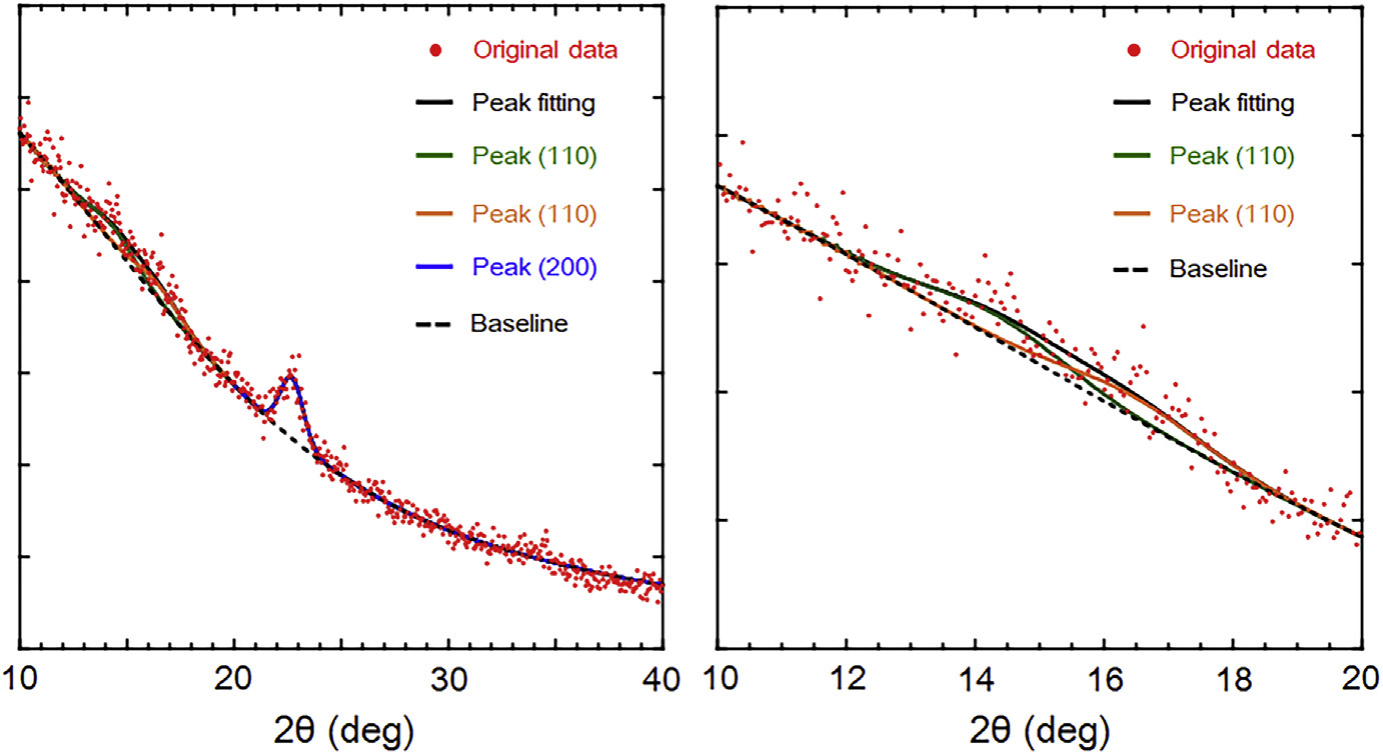
WAXD profiles of hollow-type spherical BC aerogel. Left: wide range profile, Right: enlarged profile.

The WAXD profile of HSBC gel after scCO_2_ drying is shown in Fig. 6. The scCO_2_ dried HSBC gel had a very weak crystal peak intensity because it has a small size and it is extremely light at about 80 μg. The WAXD pattern of HSBC gel had three characteristic peaks of cellulose I crystal structure at 2θ = 14.4° (1$\bar{1}$0), 16.4° (110), and 22.7° (200), and the peak of cellulose II crystal structure at 2θ = 20.1° (110) could not be confirmed (Yue et al., 2013). This result implied that the crystals in the cellulose fibrils constituting the HSBC gel were cellulose type I structure.

**Fig. 7:**
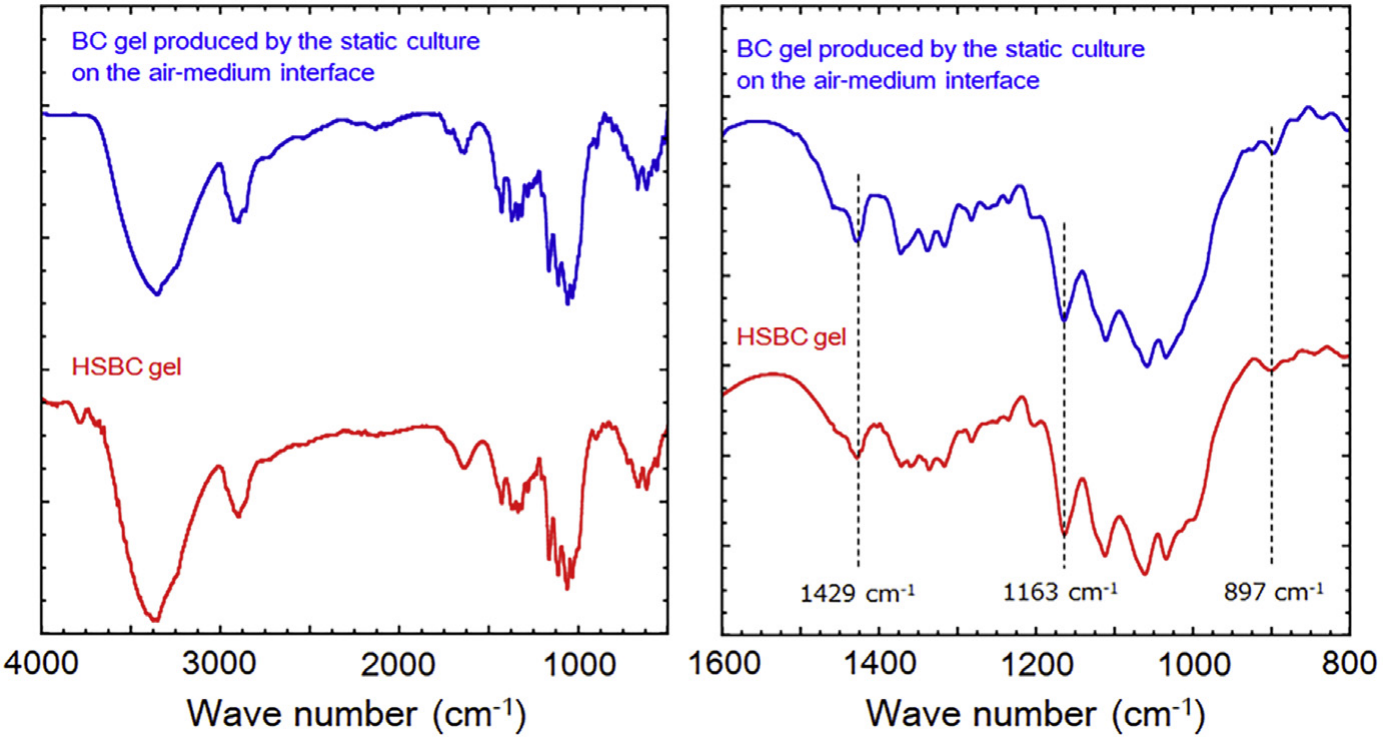
ATR-FTIR spectra of hollow-type spherical BC aerogel and conventional BC aerogel. Left: wide range spectrum, Right: enlarged spectrum.

The ATR-FTIR spectra of the HSBC gel after scCO_2_ drying is shown in Fig. 7. As a comparative sample, the BC gel obtained from the test tube was presented. It was dried by scCO_2_ in the same procedure. Both the samples showed nearly identical spectra and the absorptions at 1429 cm^−1^ (CH_2_ bending), 1163 cm^−1^ (C-O-C stretching), and 897 cm^−1^ (β-glucosidic linkage) that are specific to cellulose type I crystals (Yue et al., 2013), were confirmed.

By WAXD profile and ATR-FTIR spectra, we could confirm that the crystals in the cellulose fibrils constituting the HSBC gel were cellulose type I, which was same as that from air–medium interface.

### 3.4 Drug release behavior from HSBC gel

**Fig. 8:**
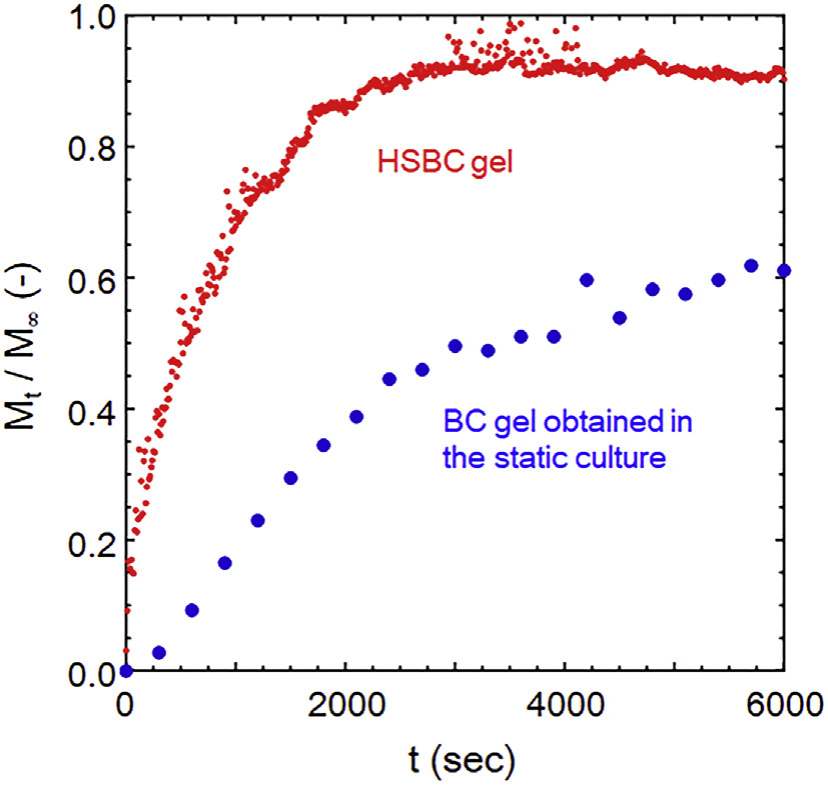
Release behavior of FITC-Dex from within the HSBC gel (diameter: 3.50 mm) and conventional BC gel. M_t_: The cumulative amount of drug released at time, M_∞_: The maximum release amount of drug.

As described above, HSBC gel is like a seamless capsule. These walls comprise of cellulose fiber networks. To investigate its feasibility as a drug delivery device, the release behavior of FITC-Dex (M_w_ = 10,000) from within the HSBC gel (diameter: 3.50 mm) was studied by UV-Vis spectroscopy. For comparison, we used the conventional BC gel obtained from the static culture, which had almost the same surface area of the HSBC gel. The HSBC gel showed rapid release initially due to a thin gelatinous membrane as compared to the conventional BC gel, and the release rate gradually decreased (Fig. 8). At 3000 seconds or more, it reached a constant value. The loading weight of FITC-Dex determined by M_∞_ was 22.6 μg.

The partition coefficient in the diffusion of dextran in the BC membrane is equal to 1 (Sokolnicki et al., 2006). The results indicate that there is no physical interaction between dextran and BC fiber such as adsorption and dextran is present in the water retained by the BC gel. The weight fraction of fibers of HSBC gel is 0.8 %; the HSBC gel is almost composed of water. The loading weight of FITC-Dex calculated from the volume of the sphere neglecting the volume of BC fiber with a significantly small amount is 22.4 μg. This calculated value is approximately equal to the experimental value. Hence, it is considered that all the FITC-Dex release occurred within about 3000 seconds.

The release behavior was analyzed using the following Korsmeyer-Peppas model Eqs. (1) and (2) (Korsmeyer et al., 1983).

(1) M_t_/M_∞_ = k t^n^ (M_t_/M_∞_ < 0.6)

(2) Log (M_t_/M_∞_) = Log k + n Log t

**Fig. 9:**
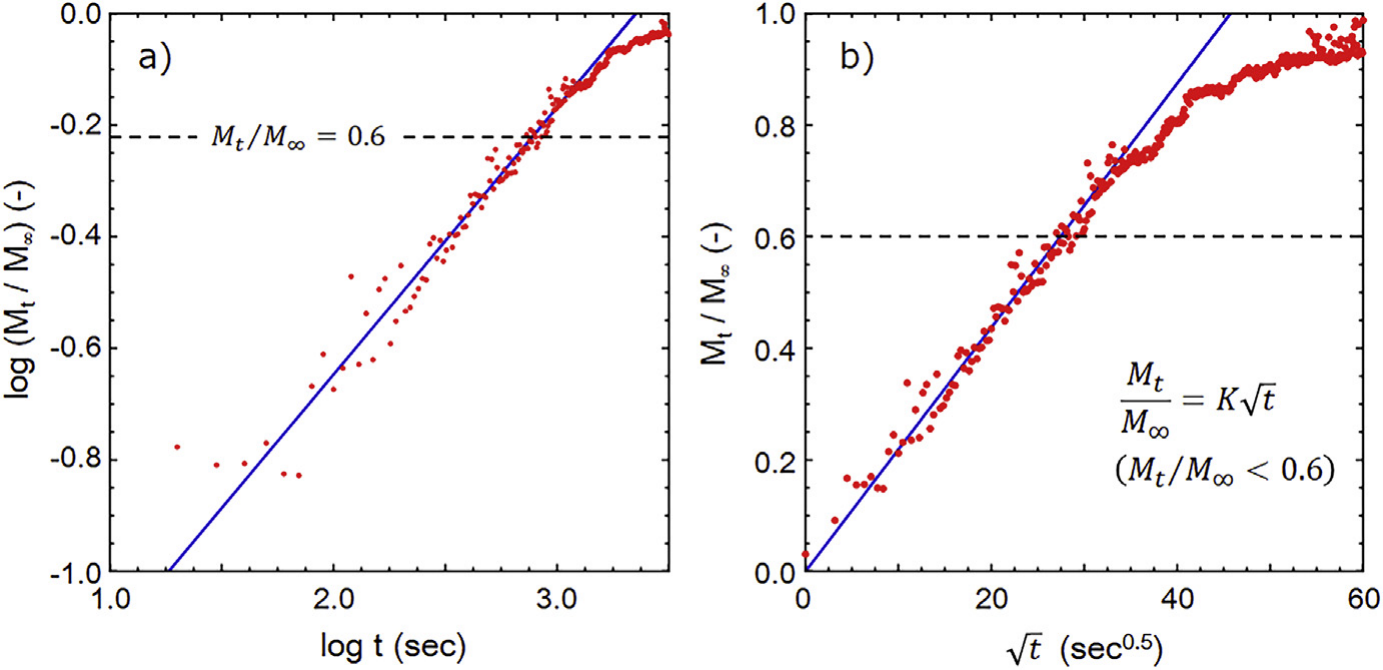
Fitting of the data of Fig. 8 to investigate the release mechanism of the HSBC gels. a) Korsmeyer-Peppas model, b) Higuchi model.

Here, M_t_ and M_∞_ are the absolute cumulative amount of drug released at time t and infinite time, respectively; k is a constant incorporating structural and geometric characteristics of the device, and n is the release exponent, indicative of the mechanism of drug release. From Eq. (2), the release exponent of the HSBC gel was n = 0.48 and k = −1.60 (Fig. 9a). If the release behavior is Fick-type, the release exponent will be n = 0.5 (Higuchi model) in the thin film and n = 0.43 in the spherical shape (Siepmann and Peppas, 2001). Additionally, R^2^ values of Korsmeyer-Peppas model and Higuchi model are 0.926 and 0.950, respectively. Release behavior of model drug from HSBC gel agrees very well with Higuchi model (Fig. 9b). Such good fitting indicates that drug release mechanism would follow Fickian diffusion for the thin films. Although it is understood that the release behavior from the HSBC gel was close to that of a thin film, since it can also be regarded as spherical anomalous diffusion (0.43 < n < 0.89) (Siepmann and Peppas, 2001), and a more detailed analysis should be necessary.

## 4 Conclusions

We developed a cultivating system for HSBC gel production without any molds or template. It consisted of floating aqueous medium droplets containing *G. xylinus* at the boundary of two non-mixed silicone oil layers with different densities. The densities of the oils used were precisely controlled to enable the floating of droplets at the boundary for cultivating periods. The size of the HSBC gel can be controlled by the volume of dropped cell suspension. After cultivation, the HSBC gels were purified and dried by supercritical drying techniques to characterize the morphology of gels. Scanning electron microscopy proved the hollow structure and well-organized fibril networks; they comprised type-I crystal structure of the cellulose by careful measurement of infra-red spectroscopy. Thus, HSBC gels have thin gelatinous membrane composed of cellulose networks, which were produced at the interface of water and oil phases. The model drug was rapidly released, and the profile agrees satisfactorily with Higuchi model, which indicates that drug release mechanism followed Fickian diffusion for the thin films.

Cellulose can modify various functional groups and functional polymers (Gonçalveset al., 2015; Alosmanov et al., 2017; Singhsa et al., 2018). Modified HSBC gels may be used as new biomedical materials such as slow drug release, controlled release by stimulation. Thus, HSBC gel would contribute towards designing new biomedical applications such as scaffolds for regenerative medicine or bio-component container or drug delivery system.

## Declarations

### Author contribution statement

Toru Hoshi: Conceived and designed the experiments; Performed the experiments; Analyzed and interpreted the data; Contributed reagents, materials, analysis tools or data; Wrote the paper.

Takao Aoyagi: Analyzed and interpreted the data; Contributed reagents, materials, analysis tools or data; Wrote the paper.

Kazuyoshi Yamazaki, Yuki Sato, and Takaya Shida: Performed the experiments.

### Funding statement

This work was supported by JSPS KAKENHI Grant Number JP15K18726.

### Competing interest statement

The authors declare no conflict of interest.

### Additional information

No additional information is available for this paper.
